# Supplementary material for: Diversity and Interactions between Picobiine Mites and Starlings
Source: Animals (Basel). 2024 Aug 29;14(17):2517. doi: 10.3390/ani14172517 (PMC11394654; doi:10.3390/ani14172517)
Supplement: Supplementary file 1 [file animals-14-02517-s001.zip › animals-3172274-Supplementary S1.pdf]

Supplementary data

Material examined:

***Picobia wisniewskii* Patan, Skoracki and Marcisova, 2024**

Material examined.

Ex. Red-winged Starling *Onychognathus morio* (Linnaeus)

Three females (NPF), three females (PF), and two males (reg. no. MS 21-1012-034); TANZANIA: Morogoro Distr., 6 May 1962, coll. Th. Andersen (host reg. no. SNSB-ZSM 64.717; female).

One female (PF) (reg. no. MS 21-1012-035); TANZANIA: Morogoro Distr., 6 May 1962 coll. Th. Andersen (host reg. no. SNSB-ZSM 63.569; male).

Two females (PF) (reg. no. MS 21-1012-032); TANZANIA: Kilimanjaro Region, Same Distr., Kilomeni, Pare Mt., coll. Th. Andersen (host in SNSB-ZSM, uncatalogued).

One female (NPF), one female (NPF), (reg. no. MS 21-1012-033); TANZANIA: Kilimanjaro Region, Same Distr., Lambeni, December 1959, coll. Th. Andersen (host in SNSB-ZSM, uncatalogued).

Two females (NPF), one female (PF), 1 male (reg. no. MS 21-1012-036); TANZANIA: Morogoro Distr., 6 May 1962, coll. Th. Andersen (host reg. no. SNSB-ZSM 63.569; male).

Ex. Bristle-crowned Starling *Onychognathus salvadorii* (Sharpe)

Two females (PF), 1 male (reg. no. MS 21-1012-037); ETHIOPIA: May 1926, coll. unknown (host reg. no. SNSB-ZSM 26.51).

***Picobia indonesiana* Skoracki and Glowska, 2008**

Material examined.

Ex. Golden Myna *Mino anais* (Lesson)

Two females (PF) and one male (reg. no. MS 21-0910-045); PAPUA NEW GUINEA: August 1910, coll. L. von Wiedenfeld (host reg. no. SNSB-ZSM 11.602; female).

Three females (PF) (reg no. MS 21-0910-048); PAPUA NEW GUINEA: July 1910, coll. L. von Wiedenfeld (host reg. no. SNSB-ZSM 11.603).

One female (NPF) (reg no. MS 21-0910-049); PAPUA NEW GUINEA: Astrolabe Bay, 10 May 1894, coll. C.B. Hagen (host in SNSB-ZSM, uncatalogued).

One female (NPF) (reg no. MS 21-0910-046); PAPUA NEW GUINEA: July 1910, coll. L. von Wiedenfeld (host reg. no. SNSB-ZSM 11.600).

Two females (PF), one male (reg no. MS 21-0910-046); PAPUA NEW GUINEA: Astrolabe Bay, Stephanest, 6 June 1894, coll. C.B. Hagen (host in SNSB-ZSM, uncatalogued).

Ex. Yellow-faced Myna *Mino dumontii* (Lesson)

One female (NPF) and one female (PF) (reg. no. MS 21-0910-050); PAPUA NEW GUINEA: 3 May 1910, coll. L. von Wiedenfeld (host in SNSB-ZSM, uncatalogued).

Ex. Asian Glossy Starling (tytleri) *Aplonis panayensis tytleri* (Hume)

Two females (NPF), one female (PF), and male (reg. no. MS 21-1012-017); INDIA: Archipelago Nicobar Islands, Kondul Isl., 13 March 1893, coll. unknown (host reg. no. SNSB-ZSM 17.644; female).

Ex. Asian Glossy Starling (sanghirensis) *Aplonis panayensis sanghirensis* (Salvadori)

Two females (PF) and two males (reg. no. MS 21-1012-018); INDONESIA: North Sulawesi Prov., Sangir Islands group, Sangir Isl., 1875, coll. Riedel (host in SNSB-ZSM, uncatalogued).

Ex. Asian Glossy Starling (strigata) *Aplonis panayensis strigata* (Horsfield)

One female (NPF), six females (PF), and one male (reg. no. MS 21-1012-019); INDONESIA: Java, West Java, Gede Mt., 3 September 1909, coll. A. Primavesi (host reg. no. SNSB-ZSM 1910/965).

Two females (NPF), three females (PF), and five males (reg. no. MS 21-1012-021); INDONESIA: Sumatra, Martin, 1894, coll. unknown (host in SNSB-ZSM, uncatalogued).

One female (NPF) and one female (PF) (reg. no. MS 21-1012-022); INDONESIA: Java, 1906, coll. Parrot (host reg. no. SNSB-ZSM A1906/100).

Two females (PF) (reg. no. MS 21-1012-023); INDONESIA: Java, West Java, Gede Mt., 26 May 1909, coll. A. Primavesi (host reg. no. SNSB-ZSM 1910/964).

Ex. Metallic Starling *Aplonis metallica* (Temminck)

One female (NPF), 2 females (PF), 2 males (reg. no. MS 21-1012-007a); PAPUA NEW GUINEA: July 1910, coll. L von Wiedenfeld (host reg. no. SNSB-ZSM 11.657; female).

One female (PF) (reg. no. MS 21-1012-008); PAPUA NEW GUINEA: 20 April 1910, coll. L von Wiedenfeld (host reg. no. SNSB-ZSM 11.658; female).

One female (PF) (reg. no. MS 21-1012-009a); PAPUA NEW GUINEA: 21 April 1910, coll. L von Wiedenfeld (host reg. no. SNSB-ZSM 11.653; female).

One female (NPF), two females (PF), and one male (reg. no. MS 21-1012-010a); PAPUA NEW GUINEA: 18 April 1910, coll. L von Wiedenfeld (host reg. no. SNSB-ZSM 11.650; female).

Two females (PF) and 1 male (reg. no. MS 21-1012-013); INDONESIA: Archipelago Raja Ampat Islands, Misool Isl., August 1911, coll. O. Tauern (host reg. no. SNSB-ZSM 14.741; male).

Three females (PF) and two males (reg. no. MS 21-1012-015a); PAPUA NEW GUINEA: Archipelago Bismarck, New Britain Isl., East New Britain Prov., Gazelle Distr., Gazelle Peninsula, coll Hahl (host reg. no. SNSB-ZSM 10.1522).

One female (PF) (reg. no. MS 21-1012-011); PAPUA NEW GUINEA: May 1910, coll. L. von Wiedenfeld (host reg. no. SNSB-ZSM 11.655).

Two females (PF), one female (NPF) (reg. no. MS 21-1012-012); INDONESIA: Archipelago Raja Ampat Islands, Misool Isl., August 1911, coll. O. Tauern (host reg. no. SNSB-ZSM 14.740; male).

One female (PF) (reg. no. MS 21-1012-014); PAPUA NEW GUINEA: June 1910, coll. L von Wiedenfeld (host reg. no. SNSB-ZSM 11.646; male).

One female (PF), 1 male (reg. no. MS 21-1012-016); PAPUA NEW GUINEA: Archipelago Bismarck, New Britain Isl. 13 July 1912, coll. Besenbruch (host reg. no. SNSB-ZSM 13.525; male).

Ex. Long-tailed Starling *Aplonis magna* (Schlegel)

Three females (NPF), five females (PF), and seven males (reg. no. MS 21-1012-024); INDONESIA: Western New Guinea, Papua Prov., Archipelago Schouten Islands, Numfor Isl., March 1873, coll. A.B. Meyer (host in SNSB-ZSM, uncatalogued).

Ex. Singing Starling *Aplonis cantoroides* (Gray)

Eight females (PF) and five males (reg. no. MS 21-1012-025); INDONESIA: Archipelago Raja Ampat Islands, Misool Isl., October 1911, coll. O. Tauern (host reg. no. SNSB-ZSM 14.737; male).

Three females (PF) (reg. no. MS 21-1012-026) from same host species; PAPUA NEW GUINEA: Archipelago Bismarck, New Britain Isl., East New Britain Prov., Gazelle Distr., Gazelle Peninsula, coll. Hahl (host reg. no. SNSB-ZSM 10.1523; male).

Three females (PF) (reg. no. MS 21-1012-027) from same host species; PAPUA NEW GUINEA: Archipelago Bismarck, New Britain Isl., East New Britain Prov., Gazelle Distr., Gazelle Peninsula, coll. Hahl (host reg. no. ZSM 10.1527).

One female (PF), one female (NPF) (reg. no. MS 21-1012-028) from same host species; PAPUA NEW GUINEA: June 1910, coll. L von Wiedenfeld (host reg. no. ZSM 11.658; female).

One female (NPF) (reg. no. MS 21-1012-029) from same host species; PAPUA NEW GUINEA: Archipelago Bismarck, New Britain Isl., March 1911, coll. Rosenbruck (host reg. no. ZSM 11.2110; female).

***Picobia lamprotornis* Klimovičová, Skoracki, Wamiti and Hromada, 2014**

Material examined.

Ex. Abbott's Starling *Poeoptera femoralis* (Richmond)

Three females (NPF), two females (PF), and three males (reg. no. MS 21-1012-042); TANZANIA: Arusha Reg., Arusha National Park, Mt. Meru, 16 November 1958, coll. v. Nagy (host reg. no. SNSB-ZSM 59.148; male).

Four females (PF) (reg. no. MS 21-1012-043); TANZANIA: Arusha Reg., Arusha National Park, Mt. Meru, 16 October 1958, coll. v. Nagy (host reg. no. SNSB-ZSM 59.147; male).

One female (PF) (reg. no. MS 21-1012-044); TANZANIA: Arusha Reg., Arusha National Park, Mt. Meru, E-Hang, 2 November 1959, coll. v. Nagy (host in SNSB-ZSM, uncatalogued; female).

One female (PF) (reg. no. MS 21-1012-045); TANZANIA: Arusha Reg., Arusha National Park, Mt. Meru, E-Hang, 28 October 1959, coll. v. Nagy (host in SNSB-ZSM, uncatalogued; female).

Ex. Kenrick's Starling *Poeoptera kenricki* Shelley

Three females (PF) and four males (reg. no. MS 21-1012-006); TANZANIA: Arusha Reg., Meru Distr., Maji ya Chai, Ngongongare, 30 December 1959, coll. unknown (host reg. no. SNSB-ZSM 60.1797; male).

Ex. Greater Blue-eared Glossy Starling *Lamprotornis chalybaeus* Hemprich and Ehrenberg

Two females (PF), one male (reg. no. MS 22-0821-005); KENYA: Mount Elgon National Park, 5 February 1925, coll. S. Alinder (host reg. no. SNSB-ZSM 26.405; female).

Three females (PF) (reg. no. MS 22-0821-001); TANZANIA: Arusha Distr., Sonja, 6 March 1911, coll. Kattwinkel (host reg. no. SNSB-ZSM 11.2751; female).

Ex. Lesser Blue-eared Glossy-Starling *Lamprotornis chloropterus* Swainson

One female (PF) (reg. no. MS 22-0821-007); TANZANIA: Ruvuma Reg., Tunduru Distr., Nandembo, 10 September 1963, coll. Th. Andersen (host reg. no. SNSB-ZSM 64.303; male).

One female (PF), one female (NPF), one male (reg. no. MS 22-0821-008); TANZANIA: Morogoro Distr., 8 July 1955, coll. Th. Andersen (host in SNSB-ZSM, uncatalogued; male).

Three females (NPF) (reg. no. MS 22-0821-009); TANZANIA: Ruvuma Reg., Tunduru Distr., Nandembo, 10 September 1963, coll. Th. Andersen (host reg. no. SNSB-ZSM 64.302; male).

Two females (NPF) (reg. no. MS 22-0821-010); TANZANIA: Mtwara Distr., Mikindani, 6 April 1966, coll. Th. Andersen (host reg. no. SNSB-ZSM 66.575; female).

Ex. Superb Starling *Lamprotornis superbus* Rüppell

Two females (NPF), one female (PF), one male (reg. no. MS 23-1120-001); KENYA: Rift Valley Region, Laikipia Distr., Laikipia Nature Conservancy (LWEC), 1 July 2013, coll. W. Wamiti (field reg. no. BB13607).

Two females (NPF) (reg. no. MS 23-1120-002); KENYA: Rift Valley Region, Laikipia Distr., Ol Pejeta Conservancy (Golf 8), 13 April 2014, coll. W. Wamiti (field reg. no. BB13669).

One female (PF) (reg. no. MS 23-1120-003); KENYA: Rift Valley Region, Laikipia Distr., Ol Pejeta Conservancy (Golf 8), 13 April 2014, coll. W. Wamiti (field reg. no. BB13664).

One female (PF) (reg. no. MS 23-1120-004); KENYA: Rift Valley Region, Laikipia Distr., Laikipia Nature Conservancy (Centre), 29 June 2013, coll. W. Wamiti (field reg. no. BB13599).

***Picobia malayi* Patan and Skoracki sp. n.**

Material examined.

Ex. Common Hill Myna *Gracula religiosa* Linnaeus

One female (NPF), four females (PF), and four males (reg. no. MS 21-0910-061); INDONESIA: Malay Archipelago, Sumatra, 1908, coll. unknown (host in SNSB-ZSM, uncatalogued).

Two females (NPF), one female (PF), and one male (reg. no. MS 21-0910-059); INDONESIA: Malay Archipelago, Sumatra, Lampung Distr., 1908, coll. W. Elbert (host in SNSB-ZSM, uncatalogued).

Two females (NPF) and one female (PF) (reg. no. MS 21-1012-089); INDONESIA: Malay Archipelago, Sumatra, 1 December 1908, coll. L. Weigant (host in SNSB-ZSM, uncatalogued).

One female (NPF) (reg. no. MS 21-1012-090); INDONESIA: Malay Archipelago, Sumatra, 3 March 1909, coll. L. Weigant (host reg. no. SNSB-ZSM 31.20).

One female (NPF) (reg. no. MS 21-0910-058); INDONESIA: Malay Archipelago, Sumatra, 1909, coll. L. Weigant (host reg. no. SNSB-ZSM 31.21).

Two females (NPF) (reg. no. MS 21-0910-060); INDONESIA: Malay Archipelago, Java, 1908, coll. W. Elbert (host in SNSB-ZSM, uncatalogued).

Ex. White-necked Myna *Streptocitta albicollis* (Vieillot)

Six females (NPF), seven females (PF) (reg. no. MS 21-0910-052); INDONESIA: Malay Archipelago, Celebes Isl., 1875, coll. Riedel (host in SNSB-ZSM, uncatalogued).

Ex. Coleto *Sacrops calvus* (Linnaeus)

One female (NPF), one female (PF) and four males (reg no. MS 21-0910-054); PHILIPPINES: Cebu Isl., 1879, coll. Burger (host reg. no. SNSB-ZSM 26.213).

One female (PF) (reg no. MS 21-0910-056); PHILIPPINES: Luzon Isl., Manila, 1963, coll. I. Marschdii (host in SNSB-ZSM, uncatalogued).

***Picobia sturni* Skoracki, Bochkov and Wauthy, 2004**

Material examined.

Ex. Common Myna *Acridotheres tristis* (Linnaeus)

Five females (PF) (reg. no. MS 21-1012-055); INDIA: Kashmir, coll. Schlagintweit (host in the SNSB-ZSM, uncatalogued).

Three female (PF) (reg. no. MS 21-1012-056); SRI LANKA: Vavuniya, 12 January 1905, coll. Doflein (host in the SNSB-ZSM, uncatalogued).

One female (PF), one female (NPF) (reg. no. MS 21-1012-057); SRI LANKA: Medawachchiya, 10 January 1905, coll. Doflein (host in the SNSB-ZSM, uncatalogued).

One female (PF) (reg. no. MS 21-1012-058); SRI LANKA: Anuradhapura, 7 January 1905, coll. Doflein (host in the SNSB-ZSM, uncatalogued).

Ex. Crested Myna *Acridotheres cristatellus* (Linnaeus)

One female (PF) (reg. no. MS 21-1012-051); CHINA: December 1912, coll. J. Gengler (host reg. no. SNSB-ZSM 28.603; male).

Ex. Jungle Myna *Acridotheres fuscus* (Wagler)

Three females (PF) (reg. no. MS 21-1012-052); NEPAL: Kathmandu Valley, Kathmandu, Baluwatar, 13 December 1964, coll. unknown (host in the SNSB-ZSM, uncatalogued).

One female (PF) (reg. no. MS 21-1012-053); NEPAL: Kathmandu, Samakhushi, 3 August 1963, coll. unknown (host in the SNSB-ZSM, uncatalogued).

Three females (PF) (reg. no. MS 21-1012-054); NEPAL: Kathmandu Valley (North), Shallaghari, 4 February 1967, coll. unknown (host reg. no. SNSB-ZSM 67.13).

Ex. Wattled Starling *Creatophora cinerea* (Meuschen)

Two females (PF) and two males (reg. no. MS 21-1012-040); KENYA: Mount Elgon National Park, Mount Elgon steppe, 8 February 1925, coll. S. Alinder (host reg. no. SNSB-ZSM 26.397; male).

Three females (PF) (reg. no. MS 21-1012-038); TANZANIA: Kilimanjaro Region, Same Distr., Lambeni, 23 December 1959, coll. Th. Andersen (host reg. no. SNSB-ZSM 61.165; female).

One female (PF) (reg. no. MS 21-1012-039); TANZANIA: Mtwara Distr., Mikindani, 13 September 1965, coll. Th. Andersen (host reg. no. SNSB-ZSM 66.577; female).

Ex. Red-billed Starling *Spodiopsar sericeus* (Gmelin)

One female (PF) (reg. no. MS 21-1012-083); CHINA: February 1912, coll. J. Gengler (host reg. no. SNSB-ZSM 28.599; male).

Three females (PF) (reg. no. MS 21-1012-084); CHINA: February 1912, coll. J. Gengler (host reg. no. SNSB-ZSM 28.600; female).

Ex. White-cheeked Starling *Spodiopsar cineraceus* (Temminck)

One female (PF) (reg. no. MS 21-1012-074); CHINA: December 1912, coll. J. Gengler (host reg. no. SNSB-ZSM 28.596; female).

Two females (PF), one female (NPF) (reg. no. MS 21-1012-075); JAPAN: expedition 1904-1905, coll. Doflein (host in SNSB-ZSM, uncatalogued; male).

Three females (PF), three females (NPF), one male (reg. no. MS 21-1012-076); JAPAN: 22 November 1912, coll. J. Gengler (host reg. no. SNSB-ZSM 28.597; male).

One female (PF) (reg. no. MS 21-1012-077); JAPAN: Honshu Isl., Yokohama, 7 December 1893, coll. unknown (host reg. no. SNSB-ZSM 17.4355; male).

Ex. Eurasian Starling *Sturnus vulgaris* Linnaeus

Four females (NPF), one female (PF) and two males (reg. no. MS 21-0910-008); KAZAKHSTAN: 11 May 1906, coll. E. Zugmayer (host in SNSB-ZSM, uncatalogued).

Two females (PF) and one male (reg. no. MS 21-0910-003); KYRGYZSTAN: Tian Schan Mts., Naryn Region, Naryn, 6 March 1908, coll. Akulin (host in SNSB-ZSM, uncatalogued).

Eight females (PF), two males (reg. no. MS 21-0910-004); KYRGYZSTAN: Tian Schan Mts., Naryn Region, Naryn, 4 March 1908, coll. Merzbacher (host in SNSB-ZSM, uncatalogued).

One female (PF) (reg. no. MS 21-0910-005); KYRGYZSTAN: Tian Schan Mts., Naryn Region, Naryn, 22 September 1908, coll. Merzbacher (host in SNSB-ZSM, uncatalogued).

Four females (PF), one female (NPF) (reg. no. MS 21-0910-006); KYRGYZSTAN: Tian Schan Mts., Naryn Region, Naryn, 12 March 1908, coll. Merzbacher (host in SNSB-ZSM, uncatalogued).

One female (PF) (reg. no. MS 21-0910-007); KYRGYZSTAN: Tian Schan Mts., Naryn Region, Naryn, 12 March 1908, coll. Merzbacher (host in SNSB-ZSM, uncatalogued).

Two females (PF) (reg. no. MS 21-0910-010); KYRGYZSTAN: Tian Schan Mts., Naryn Region, Naryn, 10 April 1910, coll. Neschivjow (host reg. no. SNSB-ZSM 17.4255).

Twelve females (NPF), two females (PF) and three males (reg. no. MS 21-0910-011); UZBEKISTAN: Tashkent, 6 March 1910, coll. Alekseev (host reg. no. SNSB-ZSM 17.4251; male).

One female (NPF), one female (PF) (reg. no. MS 21-0910-009); CHINA: Xinjiang Uyghur Autonomous Region, Kashgar, Spring 1910, coll Merzbacher (host reg. no. SNSB-ZSM 07.538).

One female (PF) (reg. no. MS 21-0920-021); ICELAND: Reykjavik, 11 December 1960, coll. A. Waag (host reg. no. IINH RM 5908, female).

Three females (PF) (reg. no. MS 21-0920-022); ICELAND: Reykjavik, 26 November 1960, coll. A. Waag (host reg. no. IINH RM 5894, female).

One female (PF) (reg. no. MS 21-0920-023); ICELAND: Reykjavik, 1 December 1960, coll. A. Waag (host reg. no. IINH RM 5903, male).

One female (PF) (reg. no. MS 21-0920-024); ICELAND: Reykjavik, 1 December 1960, coll. A. Waag (host reg. no. IINH RM 5902, female).

Three females (NPF) (reg. no. MS 21-0920-025); ICELAND: Reykjavik, 1 December 1960, coll. A. Waag (host reg. no. IINH RM 5901, female).

Two females (PF) (reg. no. MS 21-0920-026); ICELAND: Reykjavik, 26 November 1960, coll. A. Waag (host reg. no. IINH RM 5899, female).

Four females (PF), two females (NPF), one male (reg. no. MS 21-0920-027); ICELAND: Reykjavik, 1 December 1960, coll. A. Waag (host reg. no. IINH RM 5905, female).

One female (PF), one female (NPF) (reg. no. MS 21-0920-028); ICELAND: Reykjavik, 1 December 1960, coll. A. Waag (host reg. no. IINH RM 5904, female).

Two females (PF) (reg. no. MS 21-0920-029); ICELAND: Reykjavik, 15 November 1959, coll. J.B. Sigurdsson (host reg. no. IINH RM 5889, male).
Two females (PF) (reg. no. MS 21-0920-030); ICELAND: Reykjavik, 15 November 1959, coll. J.B. Sigurdsson (host reg. no. IINH RM 5888, female).
One female (NPF) and three males (reg. no. MS 21-0920-031); ICELAND: Capital Region, Kjósarhreppur, 7 March 1954, coll. A. Gardarsson (host reg. no. IINH RM 5879; female).
One female (PF) (reg. no. MS 21-0920-032); ICELAND: Reykjavik, 26 November 1960, coll. A. Waag (host reg. no. IINH RM 5896, female).
Two females (PF) (reg. no. MS 21-0920-032); ICELAND: Sílalækur, December 1954, coll. J. Andresson (host reg. no. IINH RM 5880, female).

Ex. Spotless Starling *Sturnus unicolor* Temminck
Two females (PF) (reg. no. MS 21-1012-068); ITALY: 1911, coll. P. Bonomi (host reg. no. SNSB-ZSM 17.4344; female).
One female (PF) (reg. no. MS 21-1012-070); ITALY: 1911, coll. P. Bonomi (host reg. no. SNSB-ZSM 17.4341; female).
Five females (PF), one female (NPF) (reg. no. MS 21-1012-071); ITALY: Sicci, 1906, coll. P. Bonomi (host reg. no. SNSB-ZSM 17.4345; male).
Two females (PF) (reg. no. MS 21-1012-073); ITALY: Sicci, 1906, coll. P. Bonomi (host reg. no. SNSB-ZSM 17.4347; female).
One female (NPF) and one male (reg. no. MS 21-1012-072); SPAIN: Castile and León, Salamanca, 5 January 1959, coll. H. Grin (host reg. no. SNSB-ZSM 63.167; male).
Four females (PF), two females (NPF) (reg. no. MS 21-1012-069); MOROCCO: 19 June 1909, coll. J. Gengler (host reg. no. SNSB-ZSM 28.589; female).
